# Comprehensive Analysis of Cytochrome P450 Monooxygenases Reveals Insight Into Their Role in Partial Resistance Against *Phytophthora sojae* in Soybean

**DOI:** 10.3389/fpls.2022.862314

**Published:** 2022-04-14

**Authors:** Praveen Khatri, Owen Wally, Istvan Rajcan, Sangeeta Dhaubhadel

**Affiliations:** ^1^London Research and Development Centre, Agriculture and Agri-Food Canada, London, ON, Canada; ^2^Department of Biology, University of Western Ontario, London, ON, Canada; ^3^Harrow Research and Development Centre, Agriculture and Agri-Food Canada, London, ON, Canada; ^4^Department of Plant Agriculture, University of Guelph, Guelph, ON, Canada

**Keywords:** soybean, cytochrome P450 monooxygenases, QTL, *Phytophthora sojae*, phytoalexins

## Abstract

Cytochrome P450 monooxygenases (P450) participate in the catalytic conversion of biological compounds in a plethora of metabolic pathways, such as the biosynthesis of alkaloids, terpenoids, phenylpropanoids, and hormones in plants. Plants utilize these metabolites for growth and defense against biotic and abiotic stress. In this study, we identified 346 P450 (GmP450) enzymes encoded by 317 genes in soybean where 26 *GmP450* genes produced splice variants. The genome-wide comparison of both A-type and non-A-type GmP450s for their motifs composition, gene structure, tissue-specific expression, and their chromosomal distribution were determined. Even though conserved P450 signature motifs were found in all GmP450 families, larger variation within a specific motif was observed in the non-A-type GmP450s as compared with the A-type. Here, we report that the length of variable region between two conserved motifs is exact in the members of the same family in majority of the A-type GmP450. Analyses of the transcriptomic datasets from soybean-*Phytophthora sojae* interaction studies, quantitative trait loci (QTL) associated with *P. sojae* resistance, and co-expression analysis identified some GmP450s that may be, in part, play an important role in partial resistance against *P. sojae.* The findings of our CYPome study provides novel insights into the functions of GmP450s and their involvements in metabolic pathways in soybean. Further experiments will elucidate their roles in general and legume-specific function.

## Introduction

The cytochrome P450 monooxygenases (P450s) are the heme-thiolate superfamily of enzymes that function as oxidoreductases with the heme group as a catalytic center. P450s have been reported in all the levels of taxonomical hierarchy from virus (Lamb et al., 2019) to bacteria and multicellular plants and animals along with fungi, protists, and archaea (Xu et al., 2015; Yonekura-Sakakibara et al., 2019). After the first reported plant P450 in cotton (Frear et al., 1969), they have been identified in many plant species and their diverse roles in plant development and defense mechanisms have been elucidated (Xu et al., 2015; Pandian et al., 2020). Plant P450 superfamily is divided into two clades: A-type and non-A-type. Generally, the A-type P450s are involved in plant-specific pathways, such as specialized metabolism whereas the non-A-type P450s are more diverse, and their sequences are more similar to P450s belonging to other kingdoms than to plant (Durst and Nelson, 1995; Paquette et al., 2000). Each clade is further categorized into clans that contain several P450 families. In *Arabidopsis*, the A-type P450s contain a single clan: clan71 whereas the non-A-type contains 8 clans: clan51, clan72, clan710, clan711, clan74, clan85, clan86, and clan97 (Bak et al., 2011). Plants P450 are named according to their homology and phylogenetic groupings with other known P450s (Nelson et al., 1996). Recently, advancements in sequencing technology have led to an increasing number of annotated genomes of a large number of diverse plant species. These whole genome sequence data have laid the foundation for the identification of P450 family members and elucidation of their evolutionary biology, and their possible roles in plant development and stress response. The genome-wide identification of P450s in several plant species, such as *Arabidopsis thaliana* (Bak et al., 2011), *Medicago truncatula* (Li et al., 2007), *Oryza sativa* (Wei and Chen, 2018), *Solanum lycopersicum* (Vasav and Barvkar, 2019), *Vitis vinifera* (Jiu et al., 2020), *Morus notabilis* (Ma et al., 2014), *Ricinus communis* (Kumar et al., 2014), and *Fagopyrum tataricum* (Sun et al., 2020) have been reported.

Generally, P450s contain five conserved domains: an N-terminus proline rich region (usually PPGP), an oxygen binding and activation site [I-helix (A/G)GX(E/D)T(T/S)], a K-helix consensus (EXXR), a PERF conserved sequence, and a C-terminus heme binding site (FXXGXRXCXG). Majority of plant P450s are membrane-bound proteins that are found anchored to the cytosolic side of the endoplasmic reticulum, however, they have also been found in other subcellular compartments, such as plastids (Bak et al., 2011). Majority of plant metabolic pathways contain reactions catalyzed by P450s, such as the biosynthesis of sterol, hormone, fatty acid, pigments, signaling molecules, structural polymers, phenylpropanoids, and phytoalexins (Bennett and Wallsgrove, 1994). Phytoalexins are anti-microbial compounds of different metabolic classes that are produced by plants upon biotic and abiotic stresses (Jeandet, 2015).

Soybean (*Glycine max* [L.] Merr) is one of the most important grain legumes worldwide. It is as an excellent source of protein, oil, and many specialized metabolites, such as isoflavonoids and saponins. Furthermore, it is the fourth largest principal field crop in the terms of seeded area that generates farm cash value of 2.52 billion dollars and ranks the third largest among principal field crops in Canada^1^. Canadian soybean growers encounter several challenges during soybean production, such as crop yield loss due to diseases and pests. Many efforts have been made to enhance the crop resistance to pathogens, such as diverse breeding approaches that involve introgression of resistance (R) genes from landraces to elite varieties. Although incorporating R-genes develops resistance against a specific pathogen, their monogenic effect pose selection pressure on the pathogen, and the resistance breaks down easily. Additionally, due to the high genetic diversity found in many regional population of soybean pathogens, such as *Phytophthora sojae*, R-gene mediated resistance becomes increasingly ineffective toward all populations (Dorrance et al., 2016). The oomycete *P. sojae* is a soil-borne pathogen that infects soybean plant from planting to harvest leading to a significant yield loss. Soybean cultivars resistant to *P. sojae* contain *Rps* (resistant to *P. sojae*) genes that are specific to a corresponding *Avirulence* (*Avr*) gene within *P. sojae*. Over time with selection pressure, *Avr* genes mutate resulting in soybeans containing specific *Rps* genes no longer being resistant against that specific strain of *P. sojae.* The altered pathogenic profile of *P. sojae avr* gene is referred to as pathotype, and determines which *Rps* gene that specific isolate is able to overcome (Dorrance et al., 2008). An alternate to R-gene mediated resistance is through the improvement of quantitative resistance that confers field or partial tolerance to disease. This type of tolerance is more durable and is not specific to individual pathogen strains (Pilet-Nayel et al., 2017). Quantitative resistance is governed by multiple genes with minor effects and involves plant specialized metabolism that produces a wide variety of compounds, such as Phytoalexins that help to reduce pathogen invasion or inhibit their colonization within plant cells (Kiraly et al., 2007). To date, four P450s involved in the phytoalexin glyceollin biosynthesis have been identified in soybean (Schopfer et al., 1998; Steele et al., 1999; Jung et al., 2000).

Previously, a total of 332 full-length *P450* genes and 378 pseudogenes were reported in soybean using Glyma1 assembly (Guttikonda et al., 2010). The new assembly (v4.0) has integrated a dense genetic map and corrected the major issues in pseudomolecule reconstruction that was observed in the previous assemblies. Therefore, we performed a genome-wide analysis of *P450* genes in soybean (*GmP450*) using the new soybean genome assembly, and identified 317 *GmP450* genes that encode for 346 proteins. Here, we present their phylogenetic analysis, chromosomal distribution, gene structure, and expression analysis. Furthermore, we analyzed the expression of *GmP450s* in soybean in response to *P. sojae* infection, and identified the *GmP450s* located within the quantitative trait loci (QTL) associated with partial resistance against *P. sojae.* This study lays a foundation for exploring the potential function of GmP450s and provides valuable information for breeding soybean varieties with an increased partial resistance to pathogen infection.

## Materials and Methods

### Identification and Analysis of P450s in Soybean

To identify all putative GmP450 proteins, soybean proteome (William 82 a4.v4.1) was downloaded from SoyBase^2^. A Basic Local Alignment Search Tool-Protein (BLASTP) search against the soybean proteome database using previously characterized plant P450s and known *Arabidopsis thaliana* P450s (AtP450s) obtained from the P450 database^3^ (Nelson, 2009) was performed with a threshold cutoff of *e*-value of 1e-10 and bit-score greater than 100. Furthermore, P450 sequences in different repositories, such as Pfam^4^, Phytozome 13^5^, and SoyBase (see Text Footnote 2) were also retrieved using the keyword ‘P450’ as a query. The recurring candidates were removed from the dataset and all non-overlapping GmP450s were scrutinized further. After that, the GmP450 sequences with an amino acid stretch of more than 400 were selected for further analysis. The isoelectric point (*pI*) and molecular mass of candidate GmP450s were calculated using Expasy tool^6^. The subcellular localization of GmP450s was predicted using DeepLOC server (Almagro Armenteros et al., 2017).

### Phylogenetic Analysis and Identification of Conserved Domains

Amino acid sequences of all the putative GmP450s were aligned using ClustalO (Sievers et al., 2011). Phylogenetic tree was constructed using maximum likelihood method in IQ-TREE server^7^ (Trifinopoulos et al., 2016), and the appropriate model was selected utilizing the ModelFinder method. The models JTT + F + I + G4 and LG + F + I + G4 were used, respectively, to generate the phylogenetic tree for the A-type and the non-A-type P450 groups. CYP102A1 was incorporated as an outgroup (Budde et al., 2006), and tree root was placed on it. The bootstrap value was set to 1,000 (Minh et al., 2013; Nguyen et al., 2015; Kalyaanamoorthy et al., 2017). The tree was visualized and annotated using Evolview v3 (Subramanian et al., 2019).

All the protein sequences of GmP450s were aligned with previously reported P450s from *A. thaliana*, *M. truncatula*, *Cicer arietinum, Oryza sativa, H. tuberosus, Petunia hybrid, Sorghum bicolor, Papaver somniferum*, and *Solanum lycopersicum* (Zondlo and Irish, 1999; Schoenbohm et al., 2000; Liu et al., 2003; Morikawa et al., 2006; Fang et al., 2012; Wu et al., 2013; Chang et al., 2018; Pan et al., 2018; Vasav and Barvkar, 2019) with the use of ClustalO with their individual family members separately. Aligned sequences were searched manually for the conserved P450 sequences reported earlier (Ayabe and Akashi, 2006). Amino acid spacing between the conserved domains were calculated manually.

### Gene Structure, Chromosomal Mapping, and Quantitative Trait Loci

The structure annotation of gene models (in gff3 format) of the identified *GmP450s* encompassing their gene, messenger RNA (mRNA), coding sequence (CDS), and untranslated region (UTR) coordinates were retrieved from SoyBase and fed into gene structure display server 2.0 (GSDS) (Guo et al., 2007) to display exon-intron organizations. Subsequently, bi-parental QTL for partial resistance against *P. sojae* reported in different studies were also collected from SoyBase^8^, and mapped on chromosomes along with *GmP450* genes using Mapchart v2.32. (Voorrips, 2002).

### Expression Analysis of *GmP450s*: Tissue-Specific and Upon *P. sojae* Infection

Differential expression genes (DEGs) analysis of *GmP450s* was performed using 6 publicly available RNAseq datasets of *P. sojae* infected soybean seedlings. These datasets include multiple soybean cultivars that are either resistant or susceptible to *P. sojae* infection. The RNAseq analysis was performed using CLC Genomic Workbench (Release 22.0^9^). Raw sequence reads of datasets were retrieved from the National Center for Biotechnology Information (NCBI) Sequence Read Archive (SRA) database, processed and mapped upon reference soybean genome William82 a4.v4.1. Mapping was performed using default settings by permitting two mismatches, 0.9 minimum length of fraction, and 0.8 minimum similarity fraction. Read counts were analyzed using a differential expression analysis tool within the CLC Genomic Workbench which utilizes a multi-factorial statistics based on a negative binomial Generalized Linear Model (GLM). The GLM allows to fit curves to expression values without assuming that the error on the values is normally distributed. The cutoff of fold change of 2 and 0.5 and *p* less than 0.05 was used to identify DEGs. The differential expression analysis strategy followed for different bioprojects are mentioned in Supplementary Table 1.

Tissue-specific expression patterns of all *GmP450*s were analyzed using expression data retrieved from Phytozome expression gene atlas^10^. The expression values in the form of fragments per kilobase of transcript per million mapped reads (FPKM) were collected for flower (open and unopen), root tip standard, root standard, lateral root standard, stem standard, shoot tip standard, and leaf standard tissues (Chen et al., 2020). The expression values were scaled with 2 base logarithms and normalized. Subsequently, all the transformed values were clustered hierarchically using an average linkage method and distance were calculated using the Euclidean method.

### Plant Material and Soybean–*P. sojae* Interaction

The seeds of soybean (*G. max* [L.] Merr) cultivar Misty was received from Dr. Richard Belanger, Laval University, Canada. Soybean–*P. sojae* interactions were conducted using the methods developed by Lebreton et al. (2018) with minor modifications. *P. sojae* isolates Cha-015, 1376c-004 and W6B-004 representing pathotypes *Rps*1a, 1b, 1c, 1d, 1k, 3a, 6, and 7 among the three isolates were used. Seedlings were germinated in sterile vermiculite at 27°C for 6 days under dark before transferring to the hydroponic set (Lebreton et al., 2018). Plants were grown under 12 h day length at 27°C with 450 μmoles/m^2^/s light and 18°C dark, before being inoculated with an even mixture of the three *P. sojae* isolates at a rate of 10^4^ zoospores/L of hydroponic solution or the mock inoculation of zoospore generation solution lacking the pathogen. Intact root samples were collected from the 3 biological replicates consisting of 3 root systems/replicate 24 h post-inoculation, flash frozen in liquid N_2_, and kept at −80°C. Test plants were grown for 14 days to ensure that the inoculated zoospores resulted in disease symptoms.

### RNA Extraction, Quantitative Reverse Transcription PCR

Total RNA was extracted from the roots of soybean seedlings of both *P. sojae* and mock infected (control) using the RNeasy Plant Mini Kit (Qiagen). On the column treatment of total RNA with DNase1 was performed to remove genomic DNA contamination. Total RNA (1 μg) was used for cDNA synthesis using SuperScript IV Reverse Transcriptase (Invitrogen). Quantitative real-time polymerase chain reaction (qRT-PCR) was performed using gene-specific primers (Supplementary Table 2) in three technical replicates for each sample using the SsoFast EvaGreen supermix (Bio-Rad) and CFX96 real-time PCR system (Bio-Rad). Gene expression was normalized to soybean *CONS4*. The data analysis was conducted using CFX Maestro (Bio-Rad) (Supplementary Table 3).

### Co-expression Analysis of *GmP450s*

The co-expression network of all soybean *P450*s was constructed using CoExpNetViz tool (Tzfadia et al., 2015). The normalized expression data for *GmP450s* from all the RNA-seq datasets were used to generate the network. The analysis was performed utilizing the Pearson correlation (*r*) method with lower percentile rank 5 and upper percentile rank 95 as a correlation threshold to find out the correlation between all putative *GmP450* genes. The co-expression was visualized using Cytoscape V3.8.0 (Smoot et al., 2011).

## Results

### Soybean Genome Contains 317 P450 Encoding Genes

**FIGURE 1:**
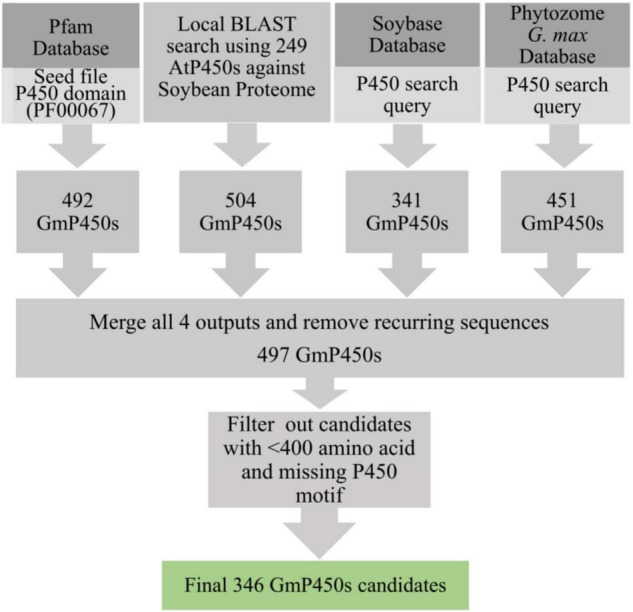
Strategy used for the identification of GmP450s. A flow diagram showing the number of hits obtained from each database search and filtering criteria that resulted into a final list of 346 putative GmP450s in soybean.

To identify all P450 encoding genes in soybean genome, we performed multiple database searches. A BLASTP search using 249 known AtP450s (Nelson et al., 2004) and 104 previously characterized P450s from different plant species as queries against the soybean proteome resulted into 504 hits. Additionally, the keyword searches using ‘P450’ as a query in Pfam, *G. max* Wm82.a4.v1 genome at Phytozome 13 and SoyBase resulted into 492, 451, and 341 hits, respectively. Eukaryotic P450s usually contain 480–560 amino acids (Danielson, 2002). The consolidation of candidate GmP450s from these searches and the subsequent removal of recurring sequences gave rise to 497 candidates which were further scrutinized for the presence of the conserved P450 domains and >400 amino acids stretch in the sequence. This process led to a total of 346 GmP450 proteins with conserved P450 domains (Figure 1).

**FIGURE 2:**
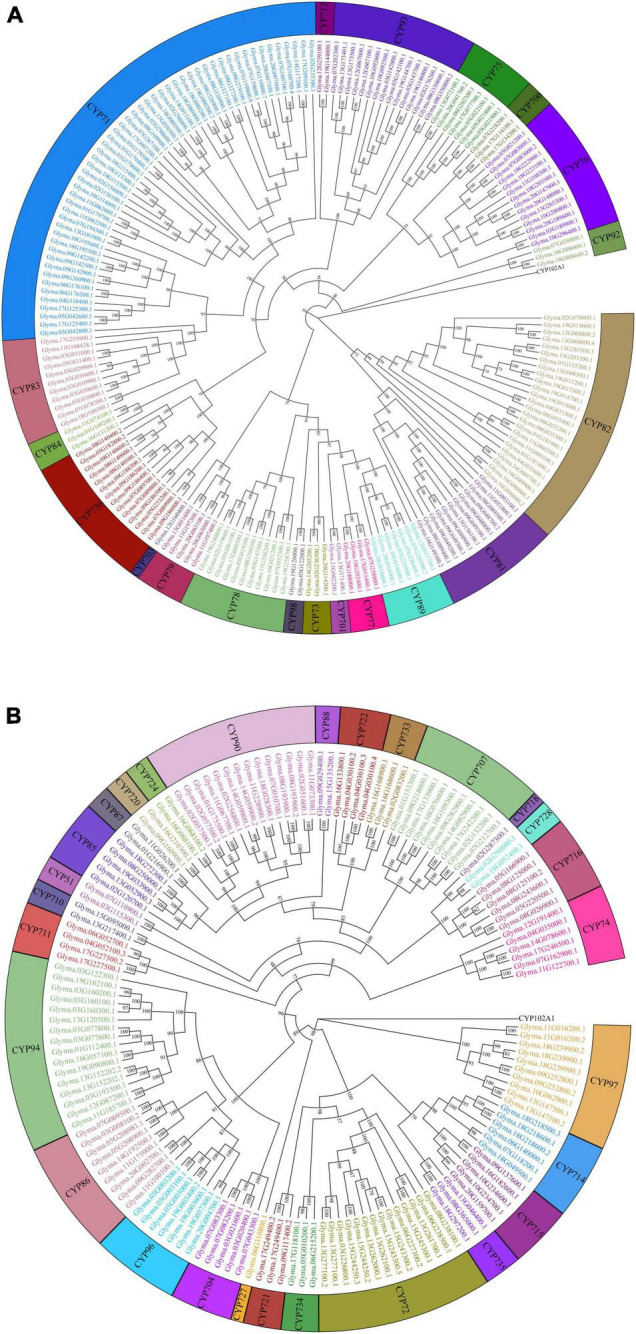
Phylogenetic analysis of GmP450s. Amino acid sequences of GmP450s were aligned in ClustalO and a phylogenetic tree was constructed using a maximum likelihood method in IQTREE2 with best fit model with a bootstrap value set to 1,000. CYP102A1 indicates an out-group used to root the tree. **(A)** A-type, and **(B)** non-A-type GmP450s.

**FIGURE 3:**
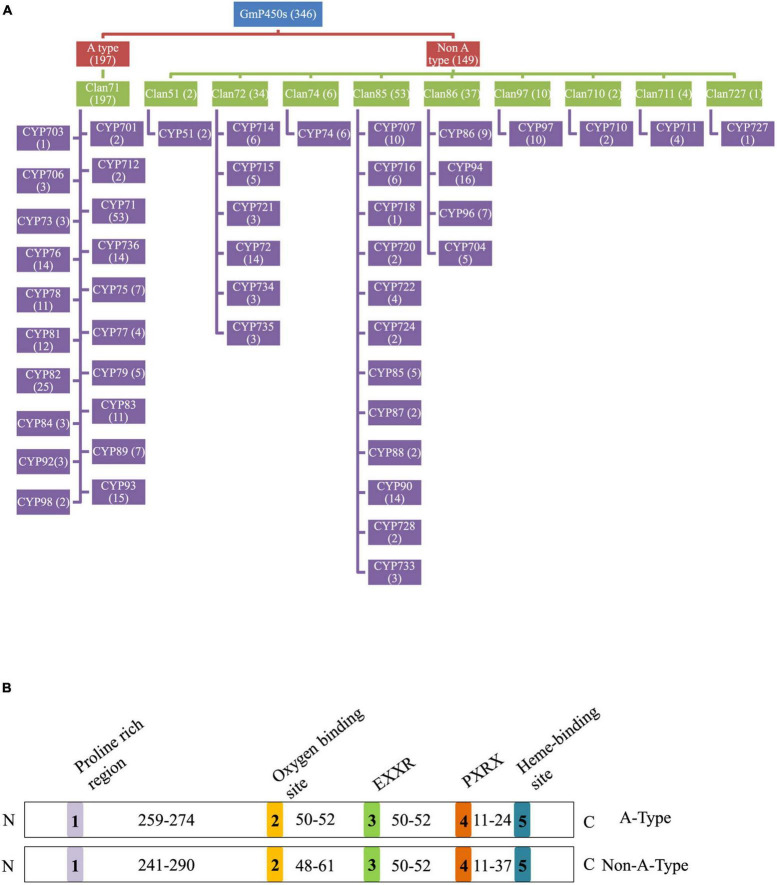
Soybean P450s. **(A)** A flowchart showing the distribution of GmP450s in clans and CYP families. GmP450 type, clans and families are color coded. The number of GmP50s under each category is indicated within the parenthesis. **(B)** Conserved motifs and number of amino acid residues in the variable regions in GmP450s. Five conserved P450 motifs are color coded and indicated by 1–5. The number of amino acid residues present in the variable region between conserved motifs is shown. N and C denotes the N-terminal and C-terminal of protein, respectively.

In total, 346 GmP450s are encoded by 317 genes where 26 of them produced multiple transcripts leading to more than one GmP450 proteins. Our search identified 10 new GmP450s compared with the previous study by Guttikonda et al. (2010). Due to the database update with reassembly and re-annotation, several discrepancies between our findings and previously reported GmP450s were observed. For example, 25 previously reported GmP450s (Guttikonda et al., 2010) are not included in our list as they either lack the P450 signature motifs or are truncated. A phylogenetic tree containing 346 candidate GmP450s along with previously reported 359 P450s from other plant species (Supplementary Table 4) grouped GmP450s into 2 groups and multiple clans (Supplementary Figure 1). A rooted phylogenetic tree was further constructed to investigate the evolutionary relationship between the GmP450 family members of the same group (Figure 2). Based on the phylogenetic analysis, GmP450s are grouped into two types: A-type with a single clan (clan71 containing 20 families) and non-A-type (9 clans and 28 families) with 197 and 149 GmP450s, respectively. Out of 48 P450 families, 3 families CYP703 (clan71), CYP718 (clan85), and CYP727 (clan727) contain only one GmP450 member. CYP71 is the largest family in soybean containing 53 members, followed by CYP82 family with 25 members (Figure 3A and Supplementary Table 4).

The calculated molecular mass of GmP450s ranged from 33.4 (CYP76; Glyma.20G147900.1) to 71.38 kDa (CYP97; Glyma.18G239900.1) with the average molecular weight of 57.7 kDa. The *pI* ranged from 4.69 (CYP97; Glyma.18G239900.2) to 9.82 (CYP728; Glyma.02G290000.1) with the average *pI* of 7.97. Majority of GmP450s were predicted to localize in the endoplasmic reticulum while some in other subcellular compartments, such as the plasma membrane, golgi complex, mitochondria, plastid, peroxisome, nucleus, and cytoplasm (Supplementary Table 4).

### Analysis of Conserved Domains and Variable Regions in GmP450s

To investigate the conserved domains in the candidate GmP450s, their protein sequences were searched for the presence of five conserved domains reported in plant P450s, such as proline-rich hinge, oxygen-binding and activation site, EXXR, PXRX, and heme-binding motif (Bak et al., 2011). The results revealed a wide variation in the conserved sequences, however, there was a consistent pattern among the family members (Supplementary Table 5). Most importantly, the K-helix (EXXR) and the three amino acid residues CXG in the heme-binding site were found conserved in the majority of GmP450 families belonging to both A-type and non-A-type, signifying their importance in enzyme activity. Furthermore, the GmP450s belonging to A-type contained less variation in the conserved motifs than non-A-type (Tables 1, 2). As shown in Table 1, in A-type, PPGP (proline-rich region) was found conserved in all the GmP450 members except for a few members of the families CYP71, CYP93, CYP701, and CYP712, where glycine at the third position in the PPGP motif was not conserved. The GmP450s belonging to the family CYP82 contained variations in proline and glycine at second and third positions, whereas most CYP78 members contained variation in all the three positions except first proline. Even though the oxygen binding domain [(A/G)GX(D/E)T(T/S)] was conserved in A-type GmP450s, variations in some amino acid residues were found (Table 1). In CYP701 family GmP450s, the first and second glycine residues are replaced with glutamic acid and threonine, respectively, while CYP78 family members contained arginine instead of glycine at the first position and valine instead of threonine or serine as the last amino acid residue. Similarly, CYP79 family GmP450s contained variations in the fifth and sixth amino acids in oxygen binding motif, where threonine or serine were replaced by asparagine and proline, respectively. Moreover, few members of CYP79 family contained variation in the second position. CYP712 and CYP84 family members showed a single variation in the oxygen binding motif. The PXRX motif is conserved in the A-type GmP450s with the amino acid residues PERF except for few members of CYP71 contained histidine instead of phenylalanine at the fourth position. Few members of CYP706, CYP736, CYP76, CYP82, and CYP84 GmP450s contained variation in glutamic acid at second position. In CYP703 and CYP79 amino acid at the fourth position is histidine instead of phenylalanine. The heme-binding sites (5th motif) are conserved in the A-type GmP450s belonging to different CYP family except for few members of CYP71, CYP76, CYP77, CYP79, CYP83, and CYP89 where glycine at end replaced by alanine (Table 1 and Supplementary Table 5). In the case of non-A type GmP450s, proline-rich region was not conserved consistently across all the CYP families (Table 2 and Supplementary Table 5). The oxygen and heme-binding sites also contained large variations possibly indicating the diversification in their functionality and activity over the time of evolution. Only the K helix and CXG in the heme-binding domain (FXXGXRXCXG) were found conserved among the non-A-type GmP450s with few exceptions in CYP72 and CYP74 families.

**TABLE 1:** Conserved motifs present in A-type GmP450 families.

GmP450 family	PPGP 1st MOTIF				(A/G)GX(E/D)T(T/S) 2nd MOTIF						EXXR 3rd MOTIF				PXRX 4th MOTIF						FXXGXRXCXG 5th MOTIF							
CYP701	P	P	V	P	E/G	T	S	D	T	T	E	X	X	R	P	E	R	F	F	G	A	G	K	R	V	C	A	G
CYP703	P	P	G	P	A	A	T	D	T	S	E	X	X	R	P	E	R	H	F	S	A	G	K	R	K	C	P	G
CYP712	P	P	S	P	A	G	T	H/E	T	S	E	X	X	R	P	E	R	F	F	G	X	G	R	R	G	C	P	G
CYP706	P	P	G	P	G	G	T	D/E	T	T/S	E	X	X	R	P	E^#^	R	F	F	G	S	G	R	R	I	C	A	G
CYP71	P	P	X	P	A/G	G	X	D/E	T	T/S	E	X	X	R	P	E	R	F*	F	G	X	G	R	R	X	C	P	G^[*dollar*]^
CYP736	P	P	G	P	A/G	A/S	X	D/E	T	T/S	E	X	X	R	P	E^#^	R	F	F	G	S	G	R	R	X	C	P	G
CYP73	P	P	G	P	A	A	I	E	T	T	E	X	X	R	P	E	R	F	F	G	V	G	R	R	S	C	P	G
CYP75	P	P	G	P	A	G	T	D	T	S	E	X	X	R	P	E	R	F	F	G	A	G	R	R	I	C	X	G
CYP76	P	P	G	P	A	G	T/I	D/E	T	T	E	X	X	R	P	E^#^	R	F	F/Y	G	X	G	R	R	X	C	X	G^[*dollar*]^
CYP77	P	P	G	P	A/G	G	T	D	T	T/S	E	X	X	R	P	E	R	F	F	G	V	G	R	R	I	C	P	G^[*dollar*]^
CYP78	P	X	X	X	R	G	T	D	T	V	E	X	X	R	P	E	R	F	F	G	X	G	R	R	V	C	P	G
CYP79	P	P	G	P	A/G	X	X	D	N	P	E	X	X	R	P	E	R	H	F	G/S	T	G	R	R	G	C	P	G^[*dollar*]^
CYP81	P	P	G	P	A/G	G	T	D/E	T/S	T/S	E	X	X	R	P	E	R	F	F	G	X	G	R	R	A	C	P	G
CYP82	P	X	X	G	A/G	A/G	X	D/E/G	T/S	X	E	X	X	R	P	E^#^	R	F	F	G/S	X	G	R	R	X	C	P	G
CYP83	P	P	G	P	A/G	A/G	T	D	T	T/S	E	X	X	R	P	E	R	F	F	G	X	G	R	R	X	C	P	G^[*dollar*]^
CYP84	P	P	G	P	G	G	T	E	T	V	E	X	X	R	P	E^#^	R	F	F	G	S	G	R	R	S	C	P	G
CYP89	P	P	G	P	A	G	T/S	D	T	T	E	X	X	R	P	E	R	F	F	G	A	G	R	R	X	C	P	G^[*dollar*]^
CYP92	P	P	G	P	G	G	T	E	S	S	E	X	X	R	P	E	R	F	F	G	A	G	R	R	M	C	P	G
CYP93	P	P	S	P	A/G	A/G	T	D/E	T/S	T/S	E	X	X	R	P	E	R	F	F	G	X	G	R	R	X	C	P	G
CYP98	P	P	G	P	A	G	M	D	T	T	E	X	X	R	P	E	R	F	F	G	X	G	R	R	V	C	P	G

*, F residue replaced by H; ^#^, E residue replaced by A/S/T/D/K; ^$^, G residue is replaced by A.

*light grey shade, conserved residues; dark grey shade, amino acid variation.*

**TABLE 2:** Conserved motifs present in non-A-type GmP450 families.

GmP450 family	PPGP 1st MOTIF				(A/G)GX(E/D)T(T/S) 2nd MOTIF						EXXR 3rd MOTIF				PXRX 4th MOTIF				FXXGXRXCXG 5th MOTIF									
CYP51	P	P	I	V	A	G	Q	H	T	S	E	X	X	R	P	D	R	F	F	G	G	G	R	H	G	C	L	G
CYP710	P	G	P	S	Q	D	A	S	T	S	E	X	X	R	P	N/D	R	F	F	G	A	G	P	H	Q	C	V	G
CYP711	P	P	G	P	A	G	S	A	T	T	E	X	X	R	P	E/D	R	F	F	G	I	G	P	R	A	C	I	G
CYP714	P	X	X	P	A	G	X	E	T/S	T/S	E	X	X	R	P	E	R	F	F	G	X	G	X	R	X	C	X	G
CYP715	P	T/S	F	P	A/G	G	H	E	T	T	E	X	X	R	P	E	R	F	F	G	F	G	G	R	M	C	V	G
CYP721	P	G/S	Y	R	A	G	K	E	T	T/S	E	X	X	R	P	M	R	F	F	G	L	G	P	R/N	I/Y	C	V	G
CYP72	P	K	R	L	A	G	Q	E/D	T/A	T/N	E	X	X	R	P	E	R	F	F	G	X	G	P	R	X	C	X	G#
CYP734	P	P	Y	R	A	G	K	Q/H	T	T	E	X	X	R	P	G	R	F	F	G	L/V	G	A/V	R	T	C	I	G
CYP735	P	K	P	C/R	A	G	H	E	T	T	E	X	X	R	P	E	R	F	F	A	S	G	P	R	N	C	V	G
CYP74	P	P	G	P	N	A/S	X	G	G	X	E	X	X	R	P$	X	R	F	P	T/S	X	X	N/D	K	Q	C	X	G#
CYP707	P	P	G	S/T	A	A	X	D	T	T	E	X	X	R	P	S	R	F	F	G	X	G	X	H/R	X	C	P	G
CYP716	P	P	G	X	A/G	G/S	H	E/D	T/S	X	E	X	X	R	P$	S/T	R	F	F	G	A/G	G	P	R	M/T	C	X	G
CYP718	P	P	G	E	A	A	H	D	T	T	E	X	X	R	P	S	R	F	F	G	G	G	P	R	V	C	A	G
CYP720	P	P	G	R	A	G	N	E	T	T	E	X	X	R	P	W	R	W	F	G	G	G	A	R	F	C	P	G
CYP722	P	P	G	N	A	G	Q	T	T	T/I	E	X	X	R	P	S/Q	R	F	F	G	M/S	G	G/P	R	T	C	L	G
CYP724	P	P	G	S	G	G	Y	E	T	T	E	X	X	R	P	F	R	W	F	G	G	G	P	R	F	C	P	G
CYP728	P	X	G	S	A	G/S	H	D	T	S	E	X	X	R	P	S	R	F	F	G	A/G	G	A/L	R/H	I/Y	C	X	G
CYP733	P	G	S	L	A	G	H	D	T	T	E	X	X	R	P	S	R	F	F	G	S	G	P	R	M	C	P	G
CYP85	P	P/Q	G	T	S	G	Y	E	T	V	E	X	X	R	P	W	R	W	F	G	G	G	T	R	Q	C	P	G
CYP87	P	P	G	S	A	S	F	E	T	T	E	X	X	R	P	W	R	W	F	G	G	G	M	R	F	C	V	G
CYP88	P	P	G	D	A	G	H	E	S	S	E	X	X	R	P	X	R	W	F	G	G	G	S	R	L	C	P	G
CYP90	P	P	G	X	A/P	G	X	E	T/S	X	E	X	X	R	P	W/G	R	W	F	G	G	G	X	R	L	C	X	G
CYP704	P	X	X	X	A	G	R/K	D	T	T/S	E	X	X	R	P	E	R	W	F	H/Q	A	G	P	R	I	C	L	G
CYP86	P	X	X	W/G	A	G	R	D	T	S	E	X	X	R	P	E	R	W	F	N	A/G	G	P	R	X	C	L	G
CYP94	P	X	X	T/Y	A	G	R/K	D	T	X	E*	X	X	R	P	X	R	W	F	Q	A	G	X	R	X	C	L	G
CYP96	P	I	I/L	G	A	G	R	E/D	T	X	E	X	X	R	P	E	R	W	F	N	A	G	P	R	X	C	L	G
CYP97	P	X	X	X	A	G	H	E	T	T/S	E	X	X	R	P	E	R	W/F	F	G/S	G	G	P	R	K	C	V	G
CYP727	P	P	S	P	H	G	C	Q	T	T	E	X	X	R	P	Y	H	F	F	G	S	G	T	R	A	C	I	G

*, E residue replaced by D; ^#^, G residue replaced by A; ^$^, P residue replaced by A or G.

*light grey shade, conserved residues; dark grey shade, amino acid variation.*

The length of the variable region between two conserved domains defines protein structure and function. Therefore, we determined the size of the variable regions in each GmP450s by measuring the distance from the end of one domain to the beginning of the adjacent domain (e.g., end of PPGP and beginning of oxygen-binding motifs, end of oxygen-binding motif to the beginning of EXXR motif, end of EXXR motif to the beginning of PXRX motif, and end of PXRX motif the beginning of heme-binding motif) and evaluated the GmP450s belonging to each family (Figure 3B). The length of the variable regions in both A- and non-A-type GmP450s were found consistent within the members of the same family (Tables 3, 4). In the majority of A-type GmP450 proteins, the length of variable region between both the oxygen binding and EXXR domains, and EXXR and PXRX domains was 51 amino acids with some exceptions (Table 3 and Supplementary Table 5). The distance between the PXRX motif and the heme-binding motif ranged from 11–24 amino acid residues in A-type GmP450s. The non-A-type GmP450s showed more variation in the length of amino acid residues (48–61 amino acids with some exceptions) between oxygen binding and EXXR motifs while majority of the non-A-type family members contained 50–52 amino acids in between EXXR and PXRX. No consistent pattern was detected in the length of the variable regions between PPGP and oxygen-binding motifs in the GmP450s belonging to both A- and non-A-type clades. Few members of the families CYP71 (Glyma.18G080200.2), CYP83 (Glyma.11G168428.1), CYP733 (Glyma.16G168600.1), CYP704 (Glyma.07G043300.1), CYP736 (Glyma.09G186000.1), and CYP724 (Glyma.16G068100.2) contained irregular spacing between the conserved motifs suggesting the possibility that they are non-functional (Supplementary Table 5).

**TABLE 3:** Length of variable regions between the conserved motifs in A-type GmP450 families.

GmP450 family	Length of variable regions between the motifs (aa)		
	2nd–3rd	3rd–4th	4th–5th
CYP701 (2)	50 (2)	51 (2)	15 (2)
CYP703 (1)	51 (1)	51 (1)	22 (1)
CYP712 (2)	51 (2)	50 (2)	13 (1), 22 (1)
CYP706 (3)	51 (2), 52 (1)	51 (3)	16 (2), 19 (1)
CYP71 (53)	23 (1), 47 (1), 50 (1), 51 (44), 52 (3), 53 (2), 55 (1)	46 (8), 50 (3), 51 (42)	16 (43), 17 (2), 21 (8)
CYP736 (14)	42 (1), 51 (12), 52 (1)	51 (7), 52 (7)	16 (14)
CYP73 (3)	50 (1), 51 (2)	51 (3)	18 (2), 21 (1)
CYP75 (7)	51 (7)	51 (7)	19 (2), 20 (5)
CYP76 (14)	51 (13), 52 (1)	51 (13), 53 (1)	16 (11), 17 (2), 18 (1)
CYP77 (4)	50 (2), 51 (2)	51 (4)	19 (2), 20 (2)
CYP78 (11)	50 (1), 51 (9), 52 (1)	48 (1), 52 (8), 53 (2)	16 (5), 17 (2), 18 (2), 20 (1), 22 (1)
CYP79 (5)	51 (5)	51 (2), 52 (3)	18 (1), 19 (3), 20 (1)
CYP81 (12)	51 (12)	51 (12)	11 (10), 12 (1), 13 (1)
CYP82 (25)	51 (23), 52 (2)	51 (21), 52 (4)	17 (3), 18 (22)
CYP83 (11)	51 (11)	50 (1), 51 (10)	16 (10), 17 (1)
CYP84 (3)	51 (3)	50 (3)	17 (3)
CYP89 (7)	52 (1), 53 (2), 54 (1), 55 (3)	50 (1), 51 (6)	18 (4), 20 (2), 24 (1)
CYP92 (3)	51 (3)	51 (3)	16 (3)
CYP93 (15)	51 (15)	50 (15)	19 (6), 20 (2), 22 (3), 23 (3), 24 (1)
CYP98 (2)	51 (2)	51 (2)	16 (2)

*2nd, oxygen binding motif; 3rd, EXXR motif; 4th, PXRX motif; 5th, heme-binding motif; aa, amino acids. The number of GmP450s is indicated in parenthesis.*

**TABLE 4:** Length of variable regions between the conserved motifs in non-A-type GmP450 families.

GmP450 family	Length of variable regions between the motifs (aa)		
	2nd–3rd	3rd–4th	4th–5th
CYP51 (2)	52 (2)	55 (2)	17 (2)
CYP710 (2)	51 (2)	49 (2)	16 (2)
CYP711 (4)	52 (4)	50 (4)	17 (4)
CYP714 (6)	50 (3), 54 (3)	51 (4), 52 (2)	16 (6)
CYP715 (5)	50 (2), 51 (3)	51 (5)	16 (5)
CYP721 (3)	51 (2), 53 (1)	51 (3)	12 (3)
CYP72 (14)	50 (14)	51 (14)	16 (14)
CYP734 (3)	51 (3)	51 (3)	16 (3)
CYP735 (3)	50 (3)	51 (3)	12 (3)
CYP74 (6)	49 (1), 52 (2), 53 (3)	49 (1), 54 (5)	21 (2), 22 (3), 27 (1)
CYP707 (10)	54 (2), 55 (3), 57 (5)	50 (10)	11 (10)
CYP716 (6)	54 (5), 55 (1)	50 (6)	12 (6)
CYP718 (1)	54 (1)	50 (1)	11 (1)
CYP720 (2)	53 (1), 54 (1)	50 (2)	19 (2)
CYP722 (4)	54 (4)	50 (4)	11 (4)
CYP724 (2)	25(1), 54 (1)	50 (2)	11 (2)
CYP728 (2)	54 (1), 55 (1)	50 (2)	13 (1), 14 (1)
CYP733 (3)	25 (1), 48 (1), 54 (1),	50 (3)	11 (3)
CYP85 (5)	54 (5)	50 (4), 51 (1)	13 (4), 14 (1)
CYP87 (2)	55 (2)	50 (2)	14 (2)
CYP88 (2)	55 (2)	50 (2)	11 (2)
CYP90 (14)	54 (3), 55 (7), 56 (4)	50 (14)	12 (3), 14 (2), 15 (3), 16 (2), 20 (1), 21 (1), 22 (2)
CYP704 (5)	60 (2), 63 (2), 78 (1)	52 (5)	16 (3), 17 (2)
CYP86 (9)	61 (5), 62 (2), 64 (1), 65 (1)	52 (9)	16 (1), 17 (2), 18 (4), 19 (2)
CYP94 (16)	45 (2), 48 (4), 50 (1), 51 (2), 52 (4), 53 (2), 54 (1)	52 (16)	13 (1), 16 (5), 21 (7), 22 (2), 23 (1)
CYP96 (7)	53 (1), 55 (6)	52 (7)	17 (7)
CYP97 (10)	50 (10)	50 (5), 51 (3), 58 (2)	18 (8), 37 (2)
CYP727 (1)	51 (1)	53 (1)	35 (1)

*2nd, oxygen binding motif; 3rd, EXXR motif; 4th, PXRX motif; 5th, heme-binding motif; aa, amino acids. The number of GmP450s is indicated in parenthesis.*

### Gene Structure Analysis and Exon-Intron Organization of *GmP450* Genes

**FIGURE 4:**
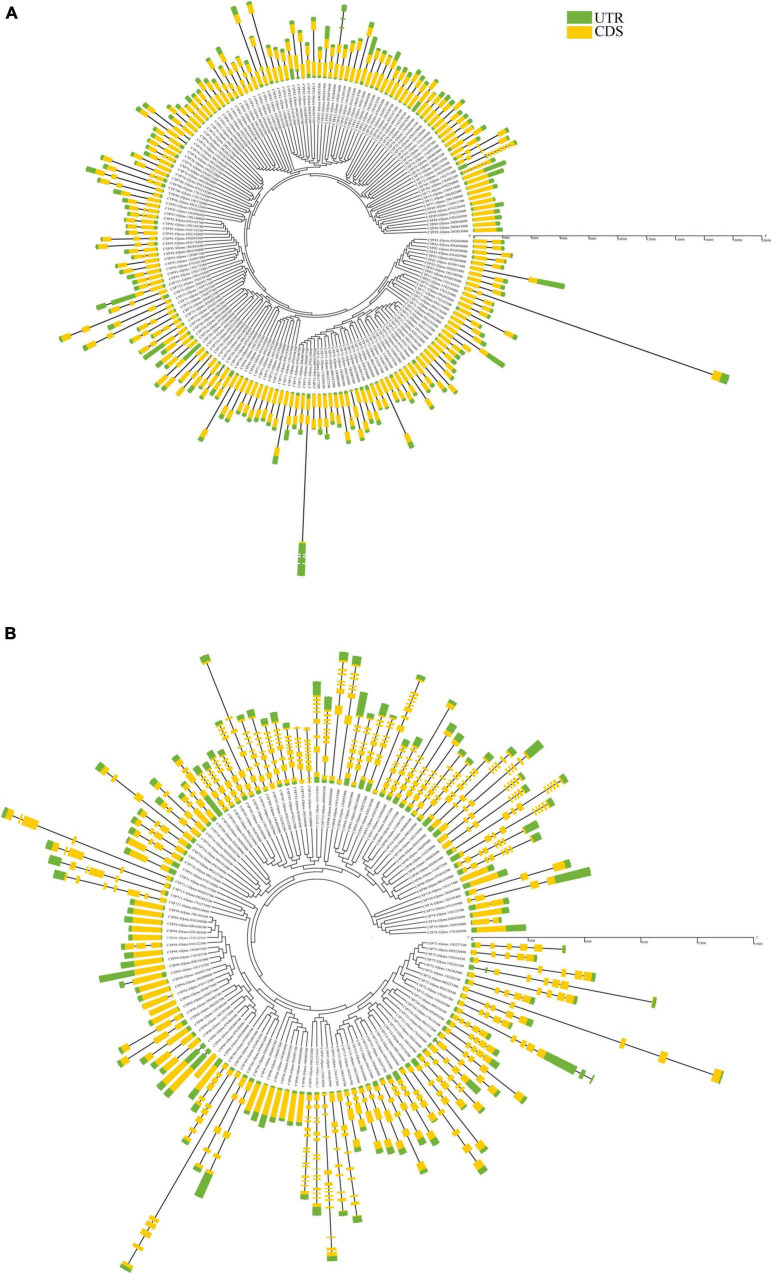
Gene structure of all putative soybean P450s showing a distinct pattern of structure among **(A)** A-type, and **(B)** non-A-type *GmP450s*. Yellow regions and black lines represent exons and introns, respectively. Green regions indicate 5′ or 3′ untranslated regions. Scale bars in base pairs are shown.

The gene structure analysis of *GmP450*s revealed a wide variation in exon-intron organization. As shown in Figure 4A, a total of 11 intronless, 136 with single intron, and 39 *GmP450s* with multiple introns with their sizes ranging from 27 (*Glyma.09G186000*) to 16,950 (*Glyma.11G168428*) nucleotides were found within the A-type. Majority of A-type CYP families contained *GmP450* genes with 1–2 introns except the two members of CYP73 (*Glyma.02G236500* and *Glyma.14G205200*) with 3 and 1 member each of CYP82 (*Glyma.16G090000*) and CYP736 (*Glyma.09G186000*) with 5 introns. All 11 members of CYP78 contained only 1 intron. Only the members of CYP701 in clan71 contained 7 introns.

On the other hand, a number of introns in *GmP450s* belonging to the non-A-type are larger as compared with A-type (Figure 4B). In clan72, most of the members of CYP72, CYP714, CYP715, CYP721, CYP734, and CYP735 are found with 3–5 introns. *Glyma.07G083200* (family CYP704) contained 5 introns where the first intron is the largest in size (909 bp) compared with other introns within the non-A-type *GmP450*s. A large variations in the intron lengths were found in the families belonging to clan 85. For example, *GmP450s* members of family CYP707, CYP722, CYP733, CYP88, CYP87, CYP90, CYP85, CYP720, and CYP724 were found with 6–8 introns except for *Glyma.04G030100* (CYP722) and *Glyma.16G168900* (CYP733) found with 9 and 11 introns, respectively. *GmP450* members of the CYP716, CYP718, and CYP728 families within clan85 contained only 2–3 introns. In clan86, apart from family CYP704 (containing 4–5 introns) 24 *GmP450s* are intronless and 7 are with 1–2 introns. *GmP450s* belonging to CYP97 family contain the largest numbers of introns ranging from 8 to 14. Furthermore, members of clan51 are found with 1 intron, clan74 with 0–3 introns, clan711 with 4–5 introns, and clan 727 with 6 introns. Clan710 *GmP450s* are found intronless.

### Tissue-Specific Transcript Abundance of *GmP450* Genes

**FIGURE 5:**
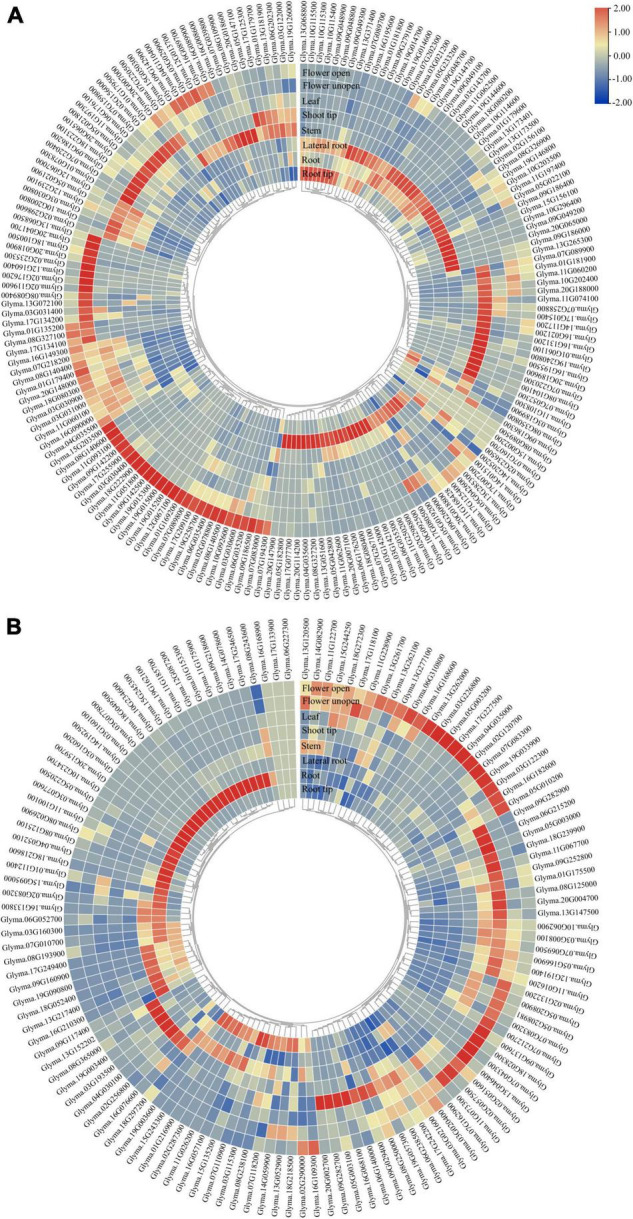
Tissue-specific expression of *GmP450* gene family. Publicly available transcriptomic data for *GmP450* genes in soybean tissues were retrieved from Phytozome13 database (https://phytozome-next.jgi.doe.gov/info/Gmax_Wm82_a4_v1) for heatmap generation. Color scale shows the range of normalized expression values in **(A)** A-type, and **(B)** non-A-type *GmP450s*.

To evaluate the expression of *GmP450* genes, we extracted the publicly available transcriptomic data from Phytozome 13 consisting of transcript levels in root tip, root, lateral root, stem, shoot tip, leaf, and flower (open and unopened). As shown in Figure 5, majority of both A- and non-A-type *GmP450s* showed tissue-specific expression pattern. A total of 65 A-type *GmP450* transcripts accumulated at higher level in root tissue, such as root tip and lateral roots compared with other tissues. Among the root-specific *GmP450*, 16 members were from family CYP71, 8 from CYP93, 7 members from CYP81, and CYP82 each, 5 from CYP736, 4 members from CYP76, 2 members each from CYP78, CYP79, one from CYP75, CYP83, CYP89, CYP92, CYP701, and CYP712. Interestingly, all the members of CYP73 were found highly expressed in root tissue. An abundance of transcript accumulation were observed in root tip for *Glyma.13G371400* belonging to the family CYP701, *Glyma.09G048800* in family CYP81, *Glyma.07G202300* and *Glyma.08G350800* in family CYP93 suggesting their roles in defense against soil borne diseases. Similar results were found in other root born disease, such as *Sclerotinia sclerotiorum* in soybean (Ranjan et al., 2019). Apart from that, *Glyma.07G218200* in family CYP706, *Glyma.06G176100, Glyma.13G181900, Glyma.16G195500, Glyma.14G117200* in family CYP71, *Glyma.12G239100* in family CYP712, *Glyma.02G236500* in family CYP73, *Glyma.09G186200* in CYP736, *Glyma.06G202300* in CYP75, *Glyma.10G200800* in CYP76, *Glyma.16G089700* in CYP82, in CYP84, *Glyma.07G220300* in CYP89 and *Glyma.12G067000* in family CYP93 found in the higher transcript abundance in aerial tissues, such as shoot tip, shoot, and leaves. Interestingly, the transcript levels of all members of CYP77 and CYP98, 6 members of CYP78, and 2 members of CYP84 (*Glyma.11G074100* and *Glyma.16G131200*) were found at much higher levels in shoot tips compared with other tissues under study. These results suggest the role of these P450s in tissue differentiation and cell wall components synthesis as some members of CYP78 were found to play a key role in regulating directional growth at the meristem and floral organogenesis (Zondlo and Irish, 1999; Eriksson et al., 2010). All members of CYP703, CYP706, and CYP83 found in higher levels in flower tissues. The FPKM counts for all *GmP450s* are shown in Supplementary Figure 2A.

The non-A-type *GmP450* transcript abundance was relatively lower in all the tissues under study compared with the A-type. Within the non-A-type *GmP450*, the members of CYP51, CYP710, CYP711, CYP87, CYP94, CYP720, CYP728, CYP721, CYP715, and CYP716 were accumulated at higher level in the root tissue while other family members showed wide variation in expression pattern. Additionally, *Glyma.19G003600* (CYP96), *Glyma.01G153300* and *Glyma.09G218600* (CYP707) showed relatively higher transcript accumulation in root tissue. All the members of CYP51 and CYP87, *Glyma.08G238100* and *Glyma.15G243300* (CYP72), and *Glyma.19G003600* (CYP96) expressed at a relatively higher level in root tips. While all the members of CYP97 showed higher expression in leaves, the non-A-type *GmP450s* with higher expression in shoot tips belonged to the families CYP707 (*Glyma.17G242200*), CYP714 (*Glyma.06G140000*), CYP716 (*Glyma.08G243600*), CYP72 (*Glyma.08G238100* and *Glyma.06G238500*), CYP74 (*Glyma.12G191400* and *Glyma.07G162900*), CYP86 (*Glyma.20G002700*), CYP88 (*Glyma.09G029400*), and CYP96 (*Glyma.05G003100*). Only a single member of CYP727 and most members of CYP704, CYP85 showed higher expression in flower. The transcript levels of *Glyma.02G132200* (CYP707), *Glyma.03G226800, Glyma.13G261700, Glyma.13G262000, Glyma.13G277100* (CYP72), *Glyma.12G191400*, *Glyma.07G162900*, *Glyma.11G122700* (CYP74), *Glyma.19G033900* (CYP85), *Glyma.18G028300, Glyma.02G051600* (CYP90), *Glyma.03G122300* (CYP94), and *Glyma.05G003200* (CYP96) also accumulated to a higher level in flower tissue. The FPKM counts for these *GmP450s* are shown in Supplementary Figure 2B.

### Chromosomal Mapping of *GmP450s* and Quantitative Trait Loci Associated With Partial Resistance Against *P. sojae*

**FIGURE 6:**
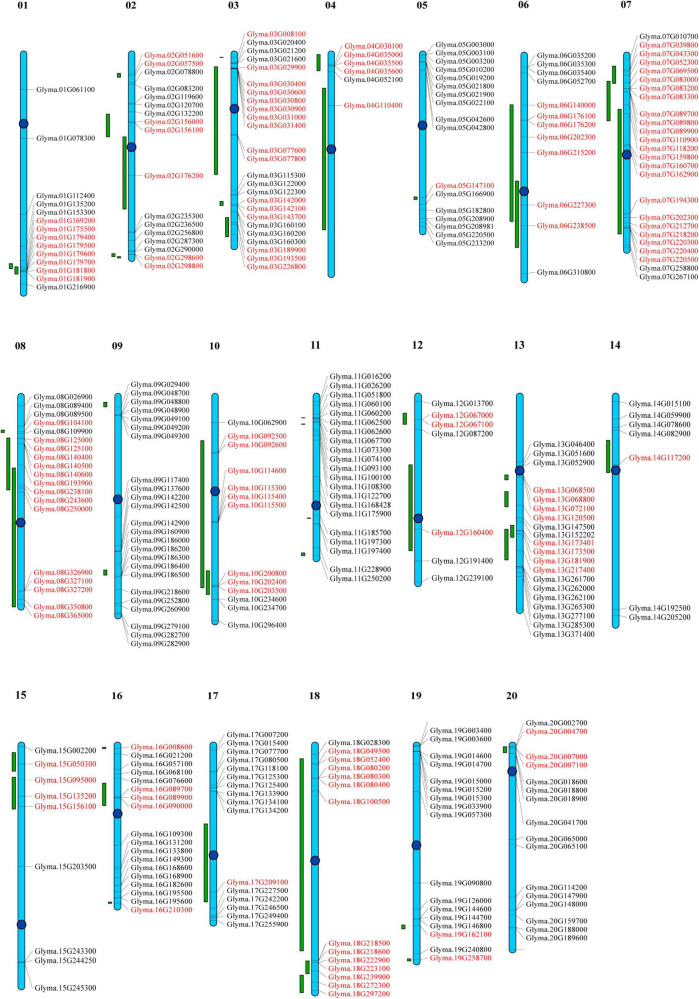
Distribution of all *GmP450* genes in soybean chromosomes and quantitative trait loci (QTL) associated with resistance against *Phytophthora sojae*. Chromosomes are drawn to scale and chromosome numbers are indicated above each chromosomes. Centromeres are indicated by blue circles. Green bars indicate QTL regions associated with resistance against *P. sojae*. Candidate *GmP450s* within the QTL region are indicated in red.

To determine the distribution of *GmP450s* on soybean chromosomes, a chromosomal map was constructed. All the 317 genes encoding 346 GmP450s are dispersed on 20 chromosomes (Supplementary Table 6). As shown in Figure 6, the *GmP450* gene density per chromosome is uneven. Chromosomes 3 and 7 contained the largest number of *GmP450* genes (25 each) followed by chromosome 9 that contained 24 *GmP450s* whereas only 6 *GmP450s* reside on chromosome 4. Most *GmP450s* were found localized toward the chromosomal ends except for some on chromosomes 7, 13, 14, and 20 that were found near the centromeres (Figure 6).

Quantitative trait loci represent a region on a chromosome associated with a quantitative trait. Several biparental QTL have been reported for partial resistance to *P. sojae*. A search for QTL linked to *P. sojae* resistance identified 188 QTL reported by 20 independent studies. These QTL were mapped on the chromosomes and *GmP450s* located within the QTL was identified. Our results revealed that a total of 130 *GmP450* genes map within the QTL regions identified for resistance against *P. sojae* (Supplementary Table 7). The QTL qHMPRR-5 on chromosome 7 contained the largest number of *GmP450s* with 11 genes. Even though chromosome 9 and 11 contained 24 and 21 *GmP450s*, respectively, none of them were found within the QTL regions. Genes within a QTL region with high phenotypic variance indicate their key role in major pathways. Our analysis revealed that QTL2-14 (Abeysekara et al., 2016), Phytoph1-2, Phytoph 2-2, Phytoph 3-2, Phytoph 4-2 (Burnham et al., 2003), Phytoph 6-2 and Phytoph 6-3 (Li et al., 2010) with a high phenotypic variance of 14.6, 15.9, 10.6, 15.9, 20.7, 21.98, and 27.98%, respectively, map on chromosome 2 that contain the genes *Glyma.02G156000*, *Glyma.02G298600, Glyma.02G298800*, and *Glyma.02G156100* (CYP71), *Glyma.02G176200* (CYP93) suggesting their possible role in resistance against *P. sojae*. Similarly, three major QTL Phytoph 2-1 (Burnham et al., 2003), Phytoph 12-1(Nguyen et al., 2012), and Phytoph14-2 (Lee et al., 2013) with 32.4, 35.8, and 16.1% phenotypic variance, respectively, mapped on chromosome 13 containing 7 *GmP450s*—*Glyma*.*13G181900, Glyma.13G068500*, and *Glyma.13G068800* (CYP82), *Glyma.13G072100* (CYP75), *Glyma.13G173500, Glyma.13G173401* (CYP93), and *Glyma.13G217400* (CYP710). Furthermore, three *GmP450s* (*Glyma.15G095000, Glyma.15G135200, and Glyma.15G156100*) on chromosome 15, *Glyma.06G202300* on chromosome 6 and three *GmP450s* (*Glyma.16G089700, Glyma.16G089900*, and *Glyma.16G090000*) on chromosome 16, four *GmP450s* (*Glyma.07G039800, Glyma.07G043300, Glyma.07G052300*, and *Glyma.07G069500*) on chromosome 7 and one *GmP450* (*Glyma.19G258700*) on chromosome 19 were mapped under major QTLs Phytoph 5-2 (Wu et al., 2011), Phytoph 6-7 (Li et al., 2010), Phytoph 8-1 (Tucker et al., 2010), QTL7-41 (Abeysekara et al., 2016), and qqQTL-19 (de Ronne et al., 2020), respectively (Supplementary Table 7 and Figure 6).

### Expression Analysis of *GmP450s* Upon *P. sojae* Infection

To identify *GmP450* genes that are induced upon *P. sojae* infection in soybean, we searched the publicly available high-throughput transcriptomic datasets for soybean–*P. sojae* interaction study. Our search identified 6 RNAseq bioprojects—PRJNA324419, PRJNA544432, PRJNA210431, PRJNA318321, PRJNA478334, and PRJNA574764 (Table 5). These datasets contained samples collected at different time points post-*P. sojae* infection from multiple soybean cultivars that are either resistant or susceptible to *P. sojae*. Two of the datasets, PRJNA478334 and PRJNA574764, were specific to the partial resistance. We included all 6 bioprojects in our analysis as the goal of the study was to identify GmP450s involved in resistance against *P. sojae* either *Rps*-mediated or quantitative. The fold change in transcript levels were calculated and genes up- or downregulated in response to *P. sojae* infection were identified. The analysis resulted in 101, 104, 93, 179, 78, and 253 differentially expressed *GmP450s* (≥2-fold change) in the datasets PRJNA324419, PRJNA544432, PRJNA210431, PRJNA318321, PRJNA478334, and PRJNA574764, respectively (Supplementary Table 1).

**TABLE 5:** List of high throughput transcriptome bioprojects used in the study.

Bioproject ID	Cultivars used	Type of tissue used	Time points hours post inoculation (hpi) or days post inoculation (dpi)	References
PRJNA324419	Williams	Hypocotyl	24 hpi	Li et al., 2016
PRJNA544432	Harosoy63 and William82	Imbibed seeds of Harosoy63 and Hairy roots of William82	24 and 48 hpi	Jahan et al., 2020
PRJNA210431	Williams and its NILs having Rps1-a, Rps1-b, 1-c and 1-k Rps3-a, 3-b, 3-c, 4, 5, and 6	Hypocotyl	24 hpi	Lin et al., 2014
PRJNA318321	Williams 82	Roots	0.5, 3, 6, and 12 hpi	Jing et al., 2016
PRJNA478334	Conrad and Sloan	Roots	24 hpi	–
PRJNA574764	PI 449459 and Misty	Roots	0, 4, 7, 14, and 21 dpi	de Ronne et al., 2020

**FIGURE 7:**
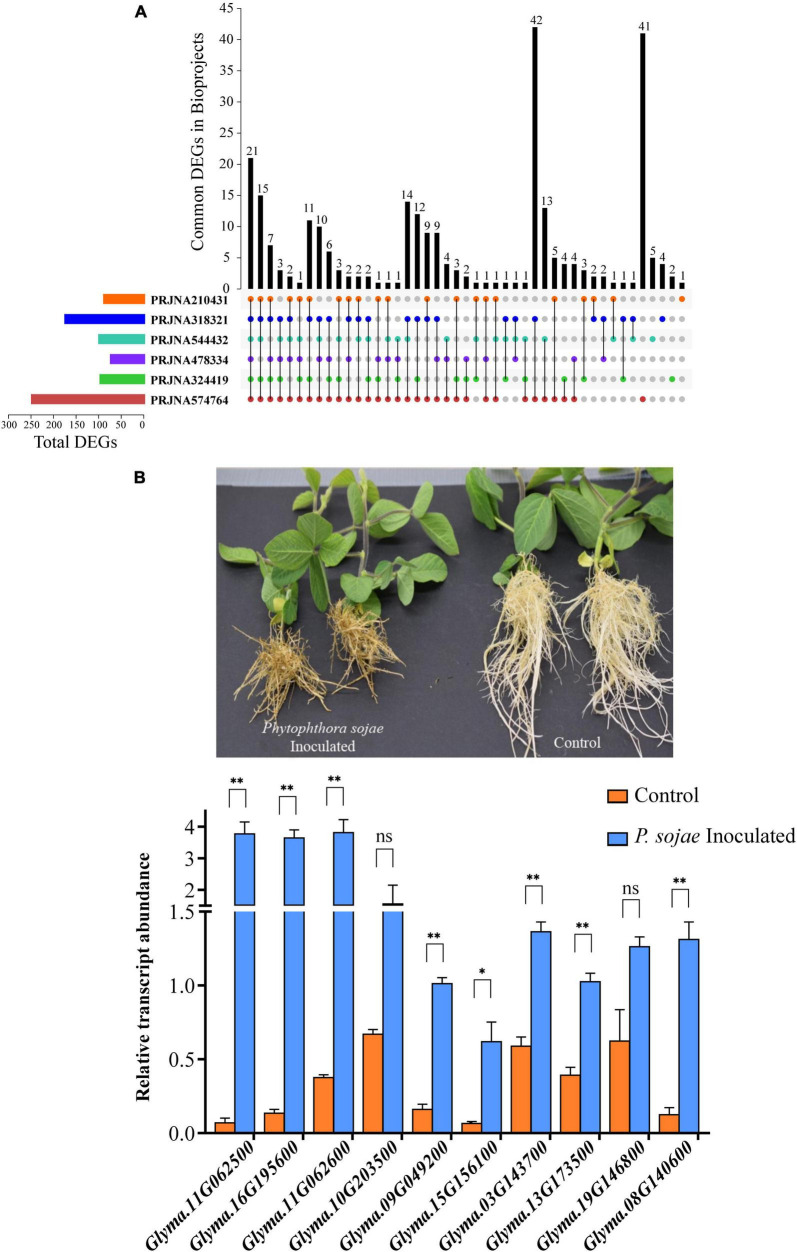
The expression analysis of *GmP450* upon exposure to *P. sojae*. **(A)** An UpSet diagram depicting the expression of *GmP450* genes that are common or unique in the bioprojects used in this study. RNAseq datasets on soybean–*P. sojae* interaction available in public domain (bioprojects: PRJNA324419, PRJNA544432, PRJNA210431, PRJNA318321, PRJNA478334, and PRJNA574764) were retrieved and differentially expressed *GmP450*s in each datasets were used in pair-wise overlap. A number of differentially expressed *GmP450*s upon *P. sojae* infection either unique to a dataset or common between the datasets are shown. **(B)** A photograph of soybean cv. Misty seedlings control (mock infected) or 21 days after *P. sojae* infection (top). The disease symptoms can be clearly seen on infected plant roots as brownish lesions. The bottom graph shows the expression analysis of ten *GmP450* genes in soybean root tissues collected after 24 h of *P. sojae* infection. cDNA was synthesized from total RNA (1 μg) from *P. sojae*-infected and control soybean roots, and qPCR was performed using gene-specific primers. Expression values were normalized against the reference gene *CONS4*. The error bars represent the SEM of two biological replicates and three technical replicates for each biological replicate. Asterisks represent the significant differences with the control samples at *p* < 0.05 (*) or *p* < 0.01 (^**^). ns indicates not significant (*p* > 0.05).

As shown in Figure 7A, 21 *GmP450s* were differentially expressed in all six transcriptome datasets used in this study indicating a strong confidence in their role in resistance against *P. sojae.* Among all the DEGs, majority of them are predicted to function in the phenylpropanoid pathway. All the differentially expressed *GmP450* genes were upregulated in response to *P. sojae* infection except for *Glyma.01G061100* (study PRJNA318321, PRJNA324419, PRJNA574764, and PRJNA210431), *Glyma.17G227500* (study PRJNA544432), *Glyma.01G061100, Glyma.05G147100*, and *Glyma.17G227500* (study PRJNA478334), *Glyma.03G142100* and *Glyma.19G014600* (study PRJNA574764, Supplementary Table 1). Out of 21 *GmP450* genes, CYP93, CYP81, and CYP82 families contained 4 each and CYP71 contained 3 *GmP450s*. Two members of CYP78 and one member each from the family CYP73, CYP76, CYP92, and CYP711 were found to be differentially expressed. Six of 21 differentially expressed *GmP450s* lie within the QTL associated with resistance to *P. sojae* where 2 genes (*Glyma.13G173500* and *Glyma.13G068800*) have been functionally characterized (Supplementary Table 1). *Glyma.13G173500* (CYP93) encodes for 2-hydroxyisoflavanone synthase (IFS2) involved in phytoalexin glyceollin biosynthesis (Steele et al., 1999) while *Glyma.13G068800* (CYP82A3) is involved in jasmonic acid and ethylene signaling pathway (Yan et al., 2016). Our results uncovered the co-localization of differentially expressed *GmP450s* in response to *P. sojae* infection and the associated QTL suggesting their role in disease resistance. The differentially expressed *GmP450* genes either unique in each dataset or common with other datasets used in this study are listed in Supplementary Table 1.

From the list of 21 genes that were common in all the bioprojects, we selected 10 *GmP450s* (such as *IFS2* and *3,9-dihydroxypterocarpan 6A-monooxygenase*) for further validation using qRT-PCR. Soybean cultivar Misty was infected with a mix culture containing multiple races of *P. sojae* and samples were collected 24 h post-infection for gene expression analysis. The effect of *P. sojae* on soybean roots and overall plant growth is clearly visible in Figure 7B. Our qRT-PCR results were consistent with those found in the transcriptome studies and revealed a significant increase in gene expression upon *P. sojae* infection. Three candidates belonging to the CYP71 family (*Glyma.11G062500, Glyma.11G062600*, and *Glyma.16G195600*) showed greater than 10-fold change upon *P. sojae* infection whereas two CYP81 candidates showed a 9.35-fold (*Glyma.15G156100*) and a 6.3-fold (*Glyma.09G049200*) increase in their expression. *Glyma.08G140600* (CYP736) expression was increased 10.5-fold in infected roots compared with control, whereas. A 2-fold increase in the expression of *Glyma.10G203500* (CYP76) and *Glyma.03G143700* and *Glyma.13G17350* (CYP93) was observed upon *P. sojae* infection (Figure 7B and Supplementary Table 3). Even though a two times higher expression of *Glyma.19G146800* (CYP93) and *Glyma.10G203500* (CYP76) was observed in the infected roots compared with control, these differences were not statistically significant.

### Co-expression Analysis of Differentially Expressed *GmP450s* in Response to *P. sojae* Infection

**FIGURE 8:**
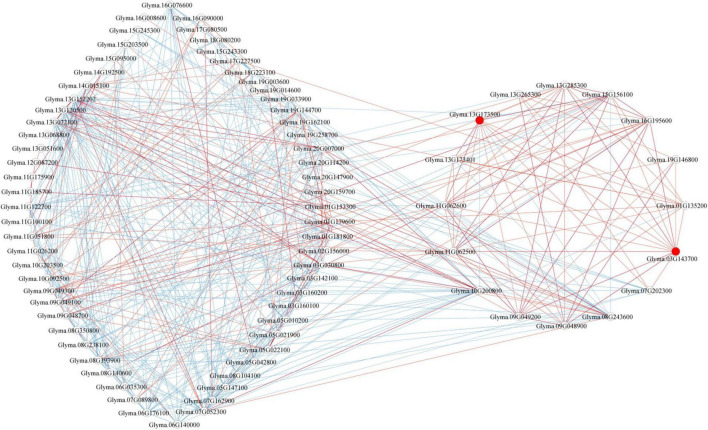
The co-expression network analysis of differentially expressed *GmP450s* in response to *P. sojae* infection. In total, 85 GmP450s differentially expressed in at least four transcriptomic studies were included in the analysis. Red and blue lines indicate positive and negative correlation, respectively. Red circles show *IFS2* (*Glyma.13G173500*) and 3*,9-dihydroxypterocarpan 6A-monooxygenase* (*Glyma.03G143700*).

Genes involved in a similar function or common metabolic pathway showed similar expression profile and exhibited similar temporal and spatial expression. Therefore, to identify the *GmP450s* involved in the resistance against *P. sojae*, we developed a co-expression network using the normalized reads of differentially expressed *GmP450* genes extracted from 6 publicly available transcriptomic datasets (Table 5). The co-expression network was developed using 85 *GmP450s* that showed differential expression in at least four transcriptomic datasets (Supplementary Table 8 and Figure 8). Out of 85 differentially expressed *GmP450* genes, 38 were located in the QTL regions associated with *P. sojae* resistance. In addition, this list contained previously characterized genes with function in stress resistance, such as *IFS2* (*Glyma.13G173500*) and 3*,9-dihydroxypterocarpan 6A-monooxygenase* (*Glyma.03G143700*). Subsequently, *IFS2* and 3*,9-dihydroxypterocarpan 6A-monooxygenase* were selected as reference points to diffuse the genes directly connected to them. Both *IFS2* and *3,9-dihydroxypterocarpan 6A-monooxygenase* are involved in glyceollin biosynthesis, therefore, as expected, they are found positively correlated with each other in the co-expression network. A total of 12 and 13 genes were identified in the individual network of *IFS2* and *3,9-dihydroxypterocarpan 6A-monooxygenase*, respectively, that belonged to 6 different GmP450 families suggesting their role in phytoalexin synthesis in soybean (Figure 8). Furthermore, *3,9-dihydroxypterocarpan 6A-monooxygenase* was found not only positively correlated with *IFS2* but also with its 9 correlated partners and also with one member each of CYP93 (*Glyma.19G146800*) and CYP76 (*Glyma.13G265300*, Supplementary Table 8). Similarly, two members each of CYP71 (*Glyma.11G062500* and *Glyma.11G062600*) and CYP82 (*Glyma.13G285300* and *Glyma.01G135200*), three members each of CYP93 (*Glyma.03G143700, Glyma.13G173401*, and *Glyma.07G202300*), and CYP81 (*Glyma.15G156100, Glyma.09G049200*, and *Glyma.09G048900*) were found positively correlated while one member each of CYP76 (*Glyma.10G200800*) and CYP716 (*Glyma.08G243600*) was found negatively correlated with *IFS2* in the co-expression analysis.

Next, we investigated the expression network of genes showing positive correlation with *IFS2* and *3,9-dihydroxypterocarpan 6A-monooxygenase*. The results revealed that the expression patterns of the 9 common genes within the *IFS2* and *3,9-dihydroxypterocarpan 6A-monooxygenase* network were positively correlated with each other. These findings strongly suggest that the genes in the *IFS2* and *3,9-dihydroxypterocarpan 6A-monooxygenase* co-expression network function in glyceollin biosynthesis in soybean.

## Discussion

In this study, we carried out a comprehensive investigation of the P450 superfamily of proteins in soybean. The genes encoding GmP450 were analyzed for their exon-intron structures, chromosomal distribution, and tissue-specific and pathogen induced gene expressions. Here, we report that soybean genome contains 317 *GmP450* genes that encode for 346 proteins of the P450 superfamily. The sequence variation observed within a conserved motif and distance between the two adjacent motifs in GmP450s are maintained within the members of a same family.

Soybean is a paleopolyploid with a genome size of 978 Mb. Although soybean has a smaller genome size compared with wheat and maize, and similar size as tomato (Supplementary Table 9), it contains larger number of P450 genes and CYP families. The discrepancy observed in the number of genes in some plant species listed in Supplementary Table 9 is due to the inclusion of P450s with less than 400 amino acid residues. A comparative analysis of genome size and the number of P450 genes in multiple plant species suggests no direct association between genome size and P450 gene number and that the number of P450s possibly depends on their functional diversity and physiological architecture. The number of P450 families and genes in each family also varies in different species. In *Arabidopsis*, 9 clans and 47 families are reported (Bak et al., 2011), while we found 10 clans and 48 families in soybean. Several earlier studies have reported the loss of some plant P450 families and gain of others (Nelson et al., 2004; Li and Wei, 2020). Similar changes are observed in soybean P450s. The loss, gain, or modification of a pre-existing molecular function leads to an adaptive evolution under a particular situation (Behe, 2010). For example: CYP92, CYP736, CYP728, CYP733, and CYP727 families are absent in *Arabidopsis* while present in soybean. On the other hand, CYP702, CYP705, CYP708, and CYP709 are absent in soybean and present in *Arabidopsis* (Supplementary Figure 1). P450s belonging to CYP92 and CYP736 families participate in homoterpene biosynthesis in maize (Richter et al., 2016) and phytoalexin biosynthesis in apple (Sircar et al., 2015), respectively. The presence of new families in soybean signifies their role in the synthesis of new compound(s) to sustain plant adaptive mechanism. CYP707 in tomato (Ji et al., 2014) and CYP88 in rice and *Arabidopsis* (Helliwell et al., 2001; Chhun et al., 2007) are reported in GA and ABA biosynthesis, respectively. In soybean, CYP722 and CYP733 clades are found closer to CYP707 and CYP88 suggesting their roles in hormone biosynthesis. However, genes belonging to the families CYP722 and CYP733 contain larger number of introns compared with the genes in CYP707 and CYP88 pointing toward the diversification of their roles possibly by duplication events.

Despite a substantial variation in GmP450 sequences, they contain a conserved P450 fold with 5 conserved motifs separated by variable regions. While the conserved motifs serve as signatures for protein function, the significance of variable regions have not been much explored. In aquaporins, a precise spacing between two NPA domains is critical for its function (Deshmukh et al., 2015). Interestingly, a specific spacing between oxygen binding and K-helix motifs, and K-helix and PXRX motifs was observed in GmP450 belonging to the same family (Tables 3, 4). The implication of the exact number of amino acids between the two motifs in GmP450 is not yet understood as this characteristic of P450 was never reported before, therefore, requires further experimental verifications. However, we speculate that it contributes to the protein folding and formation of catalytic cavities that facilitates protein–protein or protein–ligand interactions.

Plant P450s participate in the catalytic conversion of biological compounds in a plethora of metabolic pathways, such as the biosynthesis of terpenoid, alkaloid, phenylpropanoids, hormone, and xenobiotics (Nelson, 2009). These natural compounds and other defense-related proteins are produced by plants to protect them from the unfavorable conditions. *Arabidopsis* produces phytoalexin camalexin in response to the biotic and abiotic stress (Lemarie et al., 2015), while maize and cabbage synthesize zealexin (Mao et al., 2016) and cyclobrassinin (Klein and Sattely, 2015), respectively. The key P450 families participating in these phytoalexin biosynthesis belong to CYP71. Similarly, phytohormones, such as brassinosteroid, jasmonic acid, ABA, and GA also provide defense against biotic and abiotic stress (Bulgakov et al., 2019; Kohli et al., 2019; Yang et al., 2019; Wang et al., 2020) and involve P450 enzymes for their biosynthesis. The production of phytoalexin glyceollins upon exposure of soybean to environmental challenges has been long known. Silencing of *IFS* or *chalcone reductase* genes led to 90% reduction in the synthesis of glyceollin and its precursor daidzein, and suppressed hypersensitive reaction cell death leading to the loss of resistance to *P. sojae* (Graham et al., 2007). Incorporation of strong partial resistance in new elite cultivars together with the introgression of *R* genes has become a new strategy for soybean breeders to improve resistance against *P. sojae.* Partial resistance is a quantitative trait defined by a complex network that potentially include multiple mechanisms acting together or independently in a very tightly coordinated manner (Dorrance, 2018). Many QTL associated with partial resistance against *P. sojae* have been identified in soybean (Supplementary Table 7). These QTL either contain many loci across soybean genome that is associated with moderate to highly heritable resistance or with major QTL with one locus contributing to the phenotypic variance. Our effort here is to integrate multiple approaches toward identifying the sources of resistant *GmP450* allele in soybean against *P. sojae*.

Host resistance for a wide range of pathogens could be achieved by gene pyramiding. The regulatory pathways involved in the induction of phytoalexins are complex. The biosynthesis of camalexin has been shown to be affected by jasmonic acid (Rowe et al., 2010) and salicylic acid (Nawrath and Métraux, 1999) signaling pathways. Since P450s catalyze the key steps in the *de novo* synthesis of phytohormones and glyceollins in soybean, the *GmP450s* that are induced upon *P. sojae* infection and are identified in the co-expression analysis that co-localize with a QTL associated *P. sojae* resistance could be the candidates for gene pyramiding to obtain durable resistance. Both *IFS2* and *3,9-dihydroxypterocarpan 6A-monooxygenase* are involved in the glyceollin biosynthesis and co-expressed with each other along with several other uncharacterized *GmP450s.* Furthermore, some of these *GmP450* genes are located in the QTL regions reported for soybean resistance against *P. sojae*. Downstream of isoflavone aglycones synthesis reaction catalyzed by IFS, glyceollin biosynthesis involves at least additional 3 steps catalyzed by P450. The *GmP450* genes identified in our co-expression analysis are the strong candidates for functional characterization.

## Conclusion

Despite that the whole genome sequence of soybean was available since 2010 and the importance of P450s in plant metabolic pathways has been known for decades, only a handful of P450s have been functionally characterized in soybean thus far (Supplementary Table 10). Here, we identified 346 GmP450s in soybean and demonstrated for the first time that the exact length of variable region between two conserve motifs is preserved within the members of a P450 family, and speculate its possible association with protein function. The findings of the present CYPome study not only paves the way for the functional verification of GmP450s involved in soybean resistance to *P. sojae*, but also provides a well-annotated catalog of the P450s in soybean with novel insights into the functions of other GmP450s, their involvement in metabolic pathways that could be utilized for soybean breeding to improve economically important traits.

## Data Availability Statement

The original contributions presented in the study are included in the article/Supplementary Material, further inquiries can be directed to the corresponding author.

## Author Contributions

PK contributed to experimental design, collected and analyzed data, and prepared draft manuscript. OW performed soybean-*P. sojae* interaction experiment. IR contributed to manuscript preparation. SD conceived and designed the experiments, supervised all aspects of the project, and prepared the final draft manuscript. All authors contributed to the article and approved the submitted version.

## Conflict of Interest

The authors declare that the research was conducted in the absence of any commercial or financial relationships that could be construed as a potential conflict of interest.

## Publisher’s Note

All claims expressed in this article are solely those of the authors and do not necessarily represent those of their affiliated organizations, or those of the publisher, the editors and the reviewers. Any product that may be evaluated in this article, or claim that may be made by its manufacturer, is not guaranteed or endorsed by the publisher.

## Footnotes

1
https://soycanada.ca/statistics/at-a-glance/

2
https://soybase.org/

3
https://drnelson.uthsc.edu/arabidopsis/

4
http://pfam.xfam.org/family/PF00067

5
https://phytozome-next.jgi.doe.gov/

6
https://web.expasy.org/compute_pi

7
http://iqtree.cibiv.univie.ac.at/

8
https://www.soybase.org/search/qtllist_by_symbol.php

9
http://www.clcbio.com/products/clc-genomics-workbench/

10
https://phytozome-next.jgi.doe.gov/info/Gmax_Wm82_a4_v1

## Supplementary Material

Supplementary Figure 1 Phylogenetic analysis of putative GmP450. Amino acid sequences of 346 GmP450s along with *Arabidopsis* P450s and other previously characterized P450s from other plant species were aligned in ClustalO and a phylogenetic tree was constructed using a maximum likelihood method in IQTREE2 with best fit model JTT + F + I + G4 with a bootstrap value set to 1,000. The categorization of GmP450s into specific CYP family and clan was based on their grouping with the known P450s. Inner circle indicates GmP450 families while outer circle denotes clans. GmP450 families are shown by different colors for better visualization. At, *Arabidopsis thaliana*; Aa, *Anthurium amnicola*; As, *Apostasia shenzhenica*; Bv, *Beta vulgaris*; Ca, *Cicer arietinum*; Cr, *Catharanthus roseus*; CXC, *Crocosmia* × *crocosmiiflora* (montbretia); Ec, *Eucalyptus cladocalyx*; Ee, *Eustoma exaltatum*; Ga, *Gossypium arboretum*; Ge, *Glycyrrhiza echinata*; Gm, *Glycine max*; Gu, *Glycyrrhiza uralensis*; Hc, *Hibiscus cannabinus*; Ht, *Helianthus tuberosus*; Lj, *Lotus japonicas*; Md, *Malus domestica*; Me, *Manihot esculenta*; Mt, *Medicago truncatula*; Na, *Narcissus aff. pseudonarcissus*; Nf, *Nothapodytes foetida*; Os, *Oryza sativa*; Pg, *Panax ginseng*; Ph, *Petunia hybrid*; Pm, *Prunus mume*; Ps, *Papaver somniferum*; Pt, *Pinus taeda*; Sa, *Sorbus aucuparia*; Sb, *Sorghum bicolor*; Sl, *Solanum lycopersicum*; Sm, *Salvia miltiorrhiza*; Sw, *Swertia mussotii*; Va, *Vitex agnus-castus*; Vs, *Vicia sativa*; Vv, *Vitis vinifera*; Zm, *Zea mays*.

Supplementary Figure 2 **(A)** The tissue-specific expression of A-type *GmP450* genes with their fragments per kilobase of million (FPKM) map reads. Reads were normalized and scaled within a scale of −3 to +3. Individual FPKM values are mentioned in each cell. **(B)** Tissue-specific expression of non A-type GmP450 genes with their FPKM map reads. Reads were normalized and scaled within a scale of −3 to +3. Individual FPKM values are mentioned in each cell.

Supplementary Table 1 Differentially expressed GmP450 genes upon *Phytophthora sojae* infection.

Supplementary Table 2 Oligonucleotide sequences used for quantitative real-time PCR (qRT-PCR) analysis of selected soybean *GmP450s*.

Supplementary Table 3 RNA gel picture and qRT-PCR raw data.

Supplementary Table 4 Identification of GmP450s. Basic local alignment search tool (BLAST) output and characteristics of putative GmP450s.

Supplementary Table 5 The detailed analysis of conserved P450 motifs in GmP450s.

Supplementary Table 6 Distribution of *GmP450* genes in soybean chromosomes.

Supplementary Table 7 A list of quantitative trait loci (QTL) identified in soybean–*P. sojae* interactions and *GmP450s* within the QTL.

Supplementary Table 8 Co-expression analysis of differentially expressed *GmP450* genes upon *P. sojae* infection.

Supplementary Table 9 A list of genome-wide study of P450s in plant species.

Supplementary Table 10 A list of characterized P450s in soybean.
