# Supplementary figures and images for: Comprehensive Analysis of Cytochrome P450 Monooxygenases Reveals Insight Into Their Role in Partial Resistance Against Phytophthora sojae in Soybean
Source: Front Plant Sci. 2022 Apr 14;13:862314. doi: 10.3389/fpls.2022.862314 (PMC9048032; doi:10.3389/fpls.2022.862314)

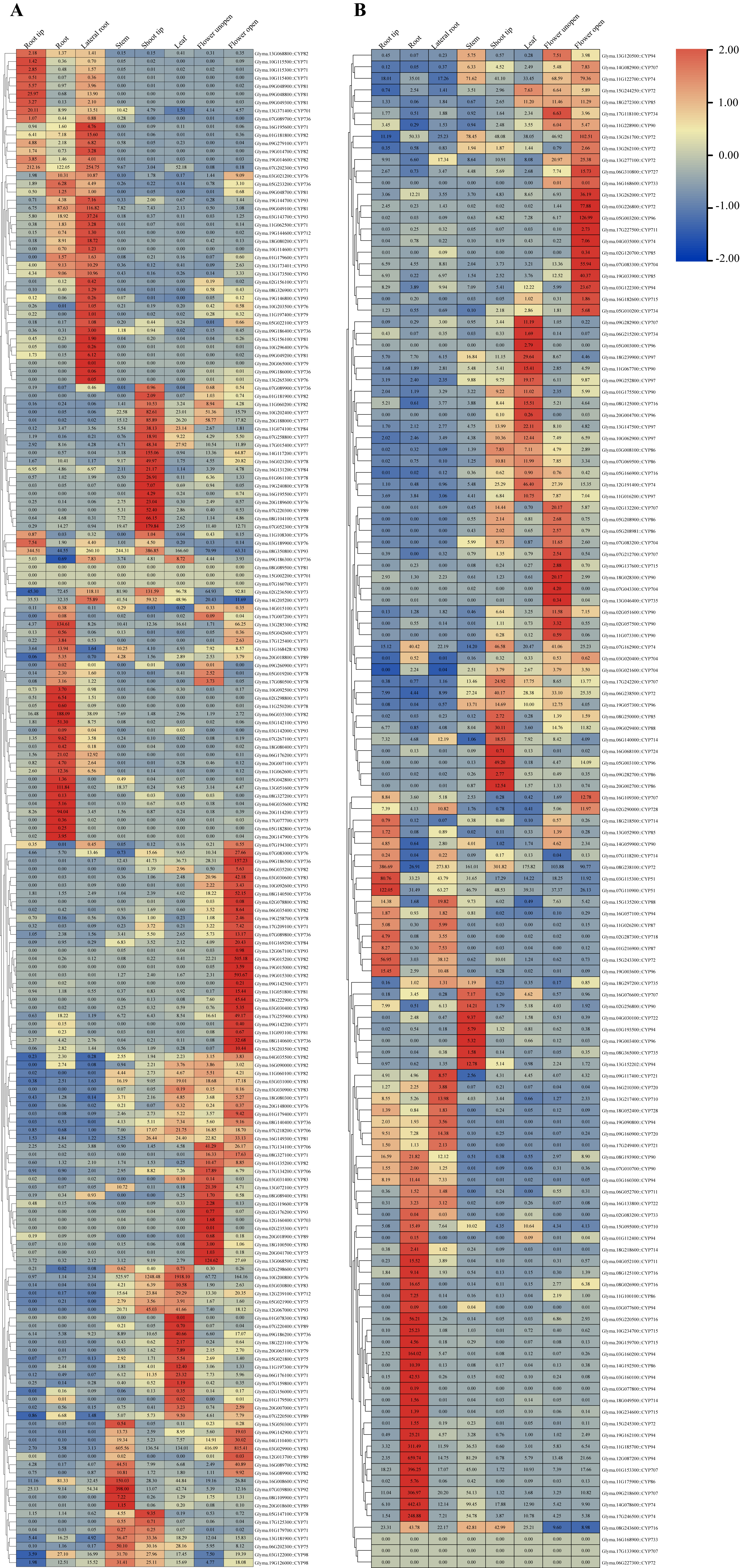

Supplement: Supplementary Figure 2 — (A) The tissue-specific expression of A-type GmP450 genes with their fragments per kilobase of million (FPKM) map reads. Reads were normalized and scaled within a scale of −3 to +3. Individual FPKM values are mentioned in each cell. (B) Tissue-specific expression of non A-type GmP450 genes with their FPKM map reads. Reads were normalized and scaled within a scale of −3 to +3. Individual FPKM values are mentioned in each cell. [file Image_2.TIF]
